# Effects of Coronavirus Disease (COVID-19) Related Contact Restrictions in Germany, March to May 2020, on the Mobility and Relation to Infection Patterns

**DOI:** 10.3389/fpubh.2020.568287

**Published:** 2020-10-08

**Authors:** Sebastian Bönisch, Karl Wegscheider, Linda Krause, Susanne Sehner, Sarah Wiegel, Antonia Zapf, Silke Moser, Heiko Becher

**Affiliations:** ^1^GIM Gesellschaft für Innovative Marktforschung mbH, Heidelberg, Germany; ^2^Institute of Medical Biometry and Epidemiology, University Medical Centre Hamburg-Eppendorf, Hamburg, Germany

**Keywords:** COVID-19, case numbers, contact restrictions, Germany, mobile tracking, mobility, interrupted time series analysis, SARIMA model

## Abstract

In an effort to contain the spread of COVID-19, Germany has gradually implemented mobility restrictions culminating in a partial lockdown and contact restrictions on 22 March. The easing of the restrictions began 1 month later, on 20 April. Analysis of the consequences of these measures for mobility and infection incidence is of public health interest. A dynamic cohort of about 2,000 individuals in Germany aged 16–89 years provided individual information on demographic variables, and their continuous geolocation via a smartphone app. Using interrupted time series analysis, we investigated mobility by age, sex, and previous mobility habits from 13 January until 17 May 2020, measured as median daily distance traveled before and after restrictions were introduced. Furthermore, we have investigated the association of mobility with the number of new cases and the reproduction number. Median daily distance traveled decreased substantially in total and homogeneously across all subgroups considered. The decrease was strongest in the last week of March followed by a slight increase. Relative reduction of mobility developed parallel with number of new cases and the daily estimated reproduction number in the weeks after contact restrictions were implemented. The increase in mobility from mid-April onwards, however, did not result in increased case numbers but in further decrease. Other behavioral changes, e.g., wearing masks, individual distancing, or general awareness of the COVID-19 hazards may have contributed to the observed further reduction in case numbers and constant reproduction numbers below one until mid-July.

## Introduction

The outbreak of coronavirus disease (COVID-19) started in China in December 2019 (1) and evolved into a pandemic affecting almost all countries worldwide. Most governments have introduced public health interventions aiming at restricting physical contact and thereby reducing transmission of the virus. The intention is to slow (or even stop) epidemic spread to lower peak health care demand (2). In Europe, Italy was the first country which was severely affected and imposed a lockdown on 22 February 2020 (3). The first COVID-19 case in Germany was reported on 24 January (4). The number of cases per day in Germany exceeded 100 on 5 March.

On 8 March, 1 week before official closure of schools on 16 March, the German Health Minister recommended to cancel events of more than 1,000 attendees. Between 12 and 18 March, all federal states successively enacted the closure of nurseries, schools, and universities. In a televised address to the nation on 16 March, Chancellor Angela Merkel urged all German citizens to reduce the spread of COVID-19 by following the imposed restrictions. In the following weeks, Germany has gradually implemented stricter mobility restrictions, culminating in a “partial lockdown” in several federal states including the introduction of contact restrictions on 22 March (5). The following 2 weeks until 5 April were denoted as lockdown period. On 20 April, the government lifted some of the restrictions. Businesses with a shop floor of up to 800 m^2^ as well as car dealers, bicycle, and bookshops were allowed to reopen. Classes leaving school this year are able to resume preparations and final examinations in school (6). Further reduction of restrictions were decided later in April and May 2020, such as opening of restaurants, children playgrounds. and others. We used the time after 26 April as representative period for relaxation. Whereas mobility restrictions were implemented mostly uniformly by federal states, there is substantial variation in the implementation provisions and timing of regulations concerning the reopening. Currently, there are increasing public debates about the appropriateness of these restrictions. Demonstrations against the restrictions increased although the majority of German citizens agree with the governmental rules at large (7). The impact of mobility restrictions critically depends on individual responses. Analyzing changes in mobility can provide insights into the degree to which interventions measures are being followed (8). Behavior changes are likely to vary between subgroups of the population. Using unique mobility data that includes individual characteristics of each person, we aim to describe changes in mobility overall and for specific subgroups of the German population from the time when the restrictions started until mid-May at a time with some shift toward normality.

## Materials and Methods

### Cohort Description

The data used in this study were contributed by members of the German online panel GapFish (9). GapFish is a professionally managed multi-purpose panel for social and consumer research. Starting from the total GapFish population, a subpanel for mobile tracking research was built by inviting panelists to install a smartphone app which continuously tracks their GPS position. This app (“Footprints App”) was provided by the Swiss market research company intervista (10) and was specially designed to continuously collect location data in a battery-friendly way. Since the sole purpose of this app is to collect location data for georeferenced research projects, installing the app has no apparent added value for the end user. Instead, the panelists receive a monthly monetary compensation for their participation in the geotracking. By installing the app, participants declare their explicit consent to being tracked for research purposes. Participation in the tracking can be canceled at any time. All data used in this study were strictly anonymous and, thus, there was no interaction between the research team and the participants. Recruitment of participants was designed to achieve a sample structure similar to the German population with respect to representative quota on age, gender, and region. Deviations from these quota were corrected by including a post-stratification weight for each participant in the analyses. When panelists decided to pause or cancel their participation in the geo tracking they were replaced by new panelists. The panel was supervised such that the number of participants per day was kept approximately constant. During the investigation period from 13 January 2020 to 17 May 2020, a daily average of 2,014 participants contributed location data, resulting in ~200,000 pairs of latitude/longitude WGS84 coordinates per day. We investigated stratification for three age groups (16–29 years, 30–59 years, ≥60 years), gender, and average mobility between 13 January and 8 March (<20 km per day, 20–50 km per day, >50 km per day). In an attempt to extend our analysis to a sub-national level, we furthermore examined the mobility patterns in three different German regions: North Rhine-Westphalia, Bavaria, and the union of the two German city-states Hamburg and Berlin. The regions were selected based on the following considerations: (i) sufficient sample size within the panel and (ii) regional variation. Berlin and Hamburg are the two largest cities in Germany with 3.8 and 1.9 million inhabitants, respectively, and also constitute federal states. Both are also preferred touristic destinations. North-Rhine Westphalia is the most populous federal state with large industrial areas in the west of Germany with some 18 million inhabitants. With more than 70,500 Sq. km, the Free State of Bavaria is the largest of the 16 federal states in Germany and is located in its southeast. With around 13 million inhabitants, it is the second most populous German state. It is also a popular touristic destination. According to^1^, since in this analysis only anonymized and grouped data were used which do not allow a re-identification of individuals an ethical statement is not required.

### Data Processing

The raw data for this study consisted of 16,730,065 time-stamped latitude/longitude WGS84 coordinate pairs and were stored and processed using the spatial database system PostGIS (11). First, we grouped the raw data by day and participant ID, resulting in daily individual (but anonymized) time-ordered tracks. We then cleaned the raw individual tracks by removing implausible data points due to signal losses, connection problems, and other sources of technical problems. The cleaning algorithm is based on the detection of sudden spatial jumps in the individual trajectories which cannot be explained by regular motion patterns. The cleaned daily individual tracks were then converted to daily individual traveled distances by simply adding the lengths of the short line segments joining two subsequent locations in the daily track of each participant. Finally, the individual daily traveled distances were aggregated by computing the daily median distance over all participants belonging to a given strata. This aggregation was carried out separately for each of the stratifications of interest.

### Interrupted Time Series Analysis

Interrupted time series analysis is a powerful methodological framework to evaluate effectiveness of health policies and interventions (12). The collected data points are split into a reference period to develop a model of the pre-intervention phase and a subsequent period for the evaluation of the changes following the intervention. In this study, the data were split into a reference (training) period from 13 January to 8 March and a post-intervention (evaluation) period from 9 March to 17 May. For the reference period a Box–Jenkins seasonal autoregressive integrated moving average model (SARIMA) was fitted to the logarithms of the median daily distances (13, 14). The SARIMA (p, d, q) × (P, D, Q, S) model is specified by seven parameters. We fixed the seasonality parameter to S = 7 due to the obvious weekly pattern. In order to find the optimal values for the remaining six parameters, we conducted a grid search guided by the Akaike Information Criterion (AIC) and examined the residual distribution and autocorrelation structure of the candidate models. The best-performing model was the SARIMA (1, 0, 0) × (2, 0, 0, 7) model. This model combines an autoregressive process of 1st order with a term modeling the effect of the day of the week. Logarithms were chosen in order to compare relative reductions and to symmetrize the model residuals. The resulting model was used as a forecast of the data to be expected during the evaluation period if no intervention was introduced and to compare these estimates with the observed data. Model training and data evaluation was performed separately for each of the three age groups and repeated for other stratifications. Based on the comparison of predicted and observed data we calculated relative reductions of median distances traveled for each day and each of the strata. For the graphical presentation, the relative reductions for 10 March (Good Friday) and 13 March (Easter Monday) were interpolated because these are public holidays in Germany and thus do not follow the usual weekly pattern. Calculations were done with Python using the module “statsmodels” (15).

### Mobility vs. Reproduction Number and Daily Number of Infections

To study the association between mobility reduction and daily numbers of infections we descriptively analyzed the time series of relative mobility reduction, daily number of cases, and governmental estimates of the reproduction number R according to newcast estimation. Data published by the Robert–Koch-Institute was used for the daily number of new infections and estimate of R in Germany^2^. The case series was smoothed using a centered 7-day moving average. The relation of the daily mobility reduction with the reproduction number over time is illustrated graphically.

## Results

The analyzed sample consists of a daily average of 2,014 participants in Germany aged 16–89 years. At the beginning of the investigation period (13 January−8 March), we observed an overall median of traveled distances measured through mobile tracking of 15.33 km. The individual distances show large variation with quartiles 3.75 km (25% quantile) and 41.25 km (75% quantile). Those values decreased considerably after mobility restrictions were implemented. Comparing the beginning of the investigation period to the period 23 March to 17 May, the median decreased 46% to 8.22 km. The quartiles decreased to 1.28 km (25% quantile) and 26.6 km (75% quantile).

**Figure 1:**
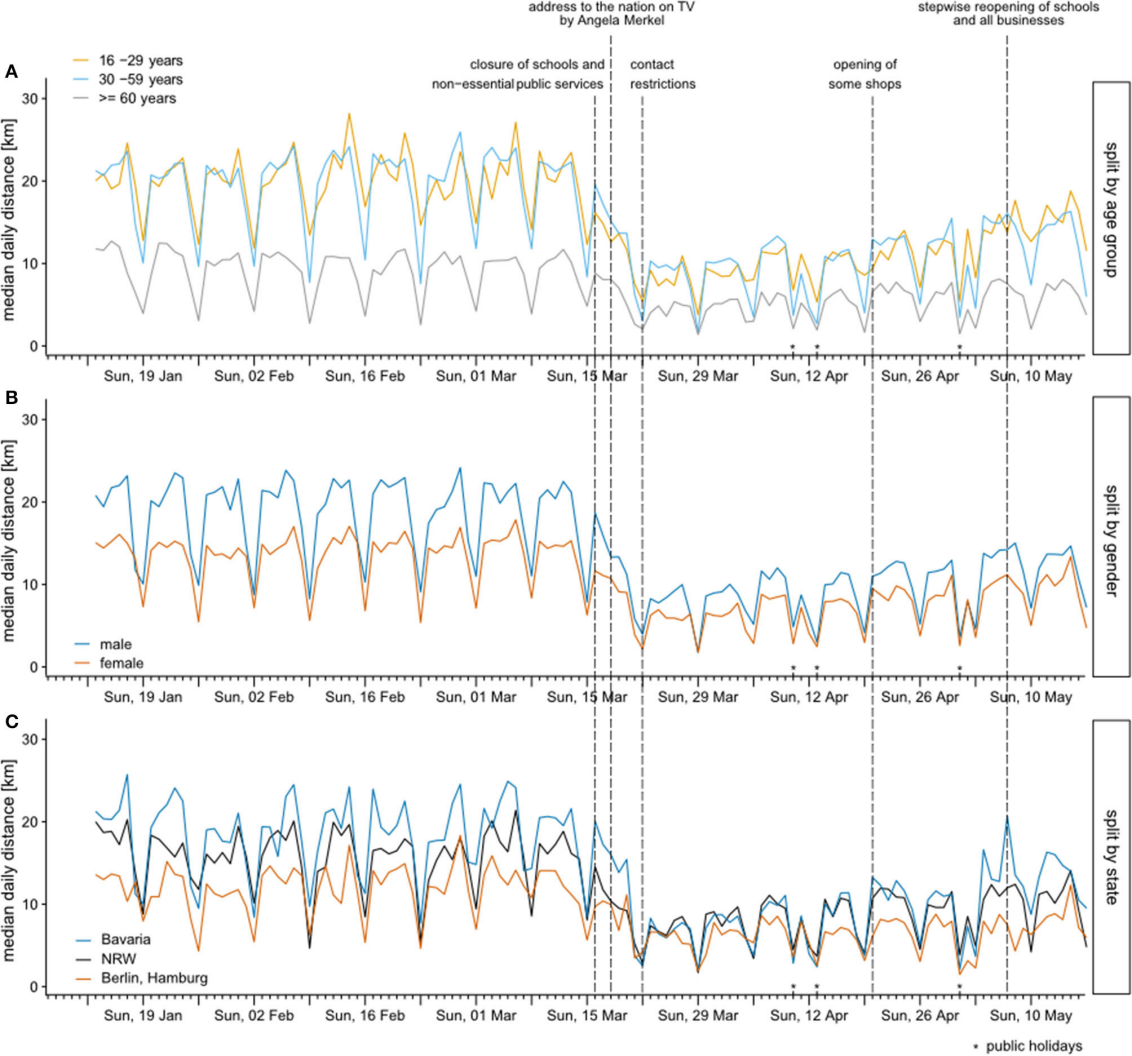
Median daily distances traveled between 13 January 2020 and 17 May 2020 stratified by three age groups (**A**), gender (**B**), and state (**C**). Dates of important governmental interventions in Germany are indicated with dashed vertical lines.

Visualizing the data in a time-resolved manner, Figure 1 shows the median distances traveled for each day during the whole study period (13 January 2020 to 17 May 2020) stratified by three age groups (Figure 1A), gender (Figure 1B), and place of residence (Figure 1C). All stratifications in Figure 1 show consistent weekly patterns for all investigated groups from beginning of the studied period (13 January 2020) until beginning of March. In addition to the dates of important governmental interventions in Germany (indicated with dashed vertical lines), 29 March with a cold spell and Good Friday (10 April) and Easter Sunday (12 April) as public holidays stand out in Figure 1 as deviations from the otherwise observed weekly patterns. The decline of distances started on the weekend 14–15 March, and is apparent for all investigated groups. While there were substantial spatial differences in mobility (with median daily distances above 20 km in Bavaria and only around 13 km in the city-states Berlin and Hamburg) before the COVID-19 outbreak in Germany, by the end of March median daily distances were almost identical across the federal states depicted in the lower panel of Figure 1. All indicated governmental interventions led to a decrease in median daily distances. A slight increase can be observed since the beginning of April and even more clearly with the relaxation of the restrictions on 20 April, but distances are still well below values before the interventions. Mobility rose substantially faster in the large federal states of Bavaria and North Rhine-Westphalia than in the city-states Berlin and Hamburg. At the end of our study period, the initial differences in mobility between states are restored, although at a smaller absolute level. Table 1 shows in detail the mean distances traveled per subgroup in the three time intervals up to 8 March (reference period), lockdown period (23 March to 5 April), and late relaxation period (26 April to 10 May). Additionally, the analysis of the average reduction rates of the two periods compared to the reference period is shown and tested, using an analysis of covariance (ANCOVA) approach. Shown is a formal comparison of the subgroup differences by an interaction test which shows that all subgroup differences were not significant. In the last two columns estimates of the reduction factors in the two periods are reported which were almost identical in the different subgroup samples. The reduction rates were ~60% in the lockdown period and still slightly over 40% in the relaxation period.

**Table 1:** Mean values and standard deviations of median daily distances for the reference period and representative periods for lockdown and relaxation within defined subgroups.

**Subgroups**	**Average sample size**	**Reference period to 08.03.2020 mean (SD)**	**Lockdown period 23.03.2020–05.04.2020 mean (SD)**	**Relaxation period 26.04.2020–10.05.2020 mean (SD)**	*p*-value group-by-period interaction^*^	**Lockdown as multiple of reference (95%-CI) *p*-value**	**Relaxation as multiple of reference (95%-CI) *p*-value**
**Gender**							
Female	930	13.5 (±3.1)	5.7 (±1.6)	8.3 (±2.7)	0.939	0.41 (0.35, 0.47) <0.001	0.57 (0.50, 0.66) <0.001
Male	1084	18.8 (±4.6)	7.8 (±2.2)	10.9 (±3.7)			
**Age groups**							
16–29 years	431	20.0 (±3.4)	8.2 (±1.6)	12.7 (±3.0)	0.744	0.43 (0.38, 0.49) <0.001	0.59 (0.53, 0.67) <0.001
30–59 years	1,283	19.6 (±4.6)	8.5 (±2.8)	11.9 (±4.1)			
≥60 years	300	9.2 (±2.7)	4.3 (±1.2)	5.5 (±2.2)			
**Region**							
Bavaria	280	18.5 (±4.6)	6.8 (±2.0)	11.1 (±4.7)	0.202	0.42 (0.37, 0.48) <0.001	0.53 (0.47, 0.61) <0.001
Berlin/Hamburg	165	11.9 (±2.9)	5.8 (±1.5)	6.2 (±2.4)			
North Rhine-Westphalia	480	15.8 (±3.7)	7.0 (±2.2)	9.4 (±2.9)			

* *Test of the period-by-subgroup interaction and reported global period effects with corresponding 95%-confidence intervals (CI) are based on the average logarithmized daily median distances*.

**Figure 2:**
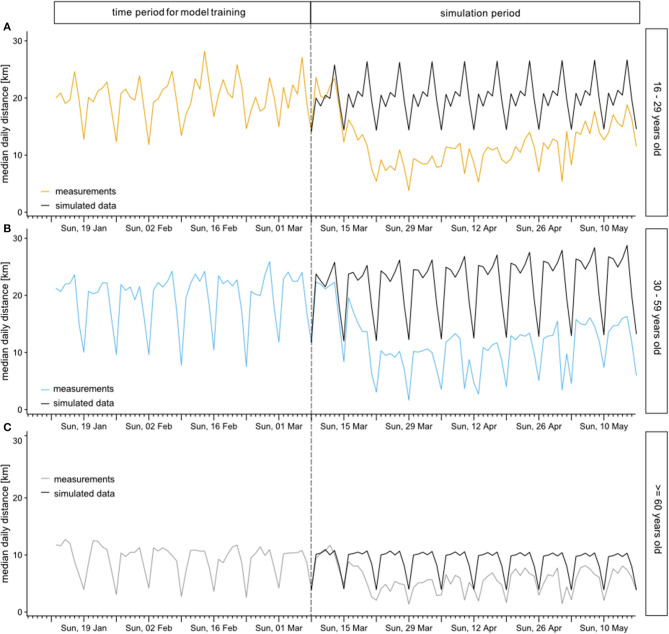
Observed median daily distances traveled (colored) by age group for the total period and simulated time series during the simulation period (black). Considered age groups were 16-29 years **(A)**, 30-59 years **(B)**, and 60 years and older **(C)**.

To estimate relative reduction of median daily distances, we first used an interrupted time series approach (12). Figure 2 shows the observed median daily distances traveled by age group for the total period and the projections in the simulation period resulting from the time series model. When comparing observed median daily distances to simulation results, the median daily distance decreases distinctively for all age groups, with the highest absolute reduction for the younger age groups (Figure 2A). Individuals 60 years and older have a smaller absolute reduction, however also a lower median daily distance before the restrictions (Figure 2C).

**Figure 3:**
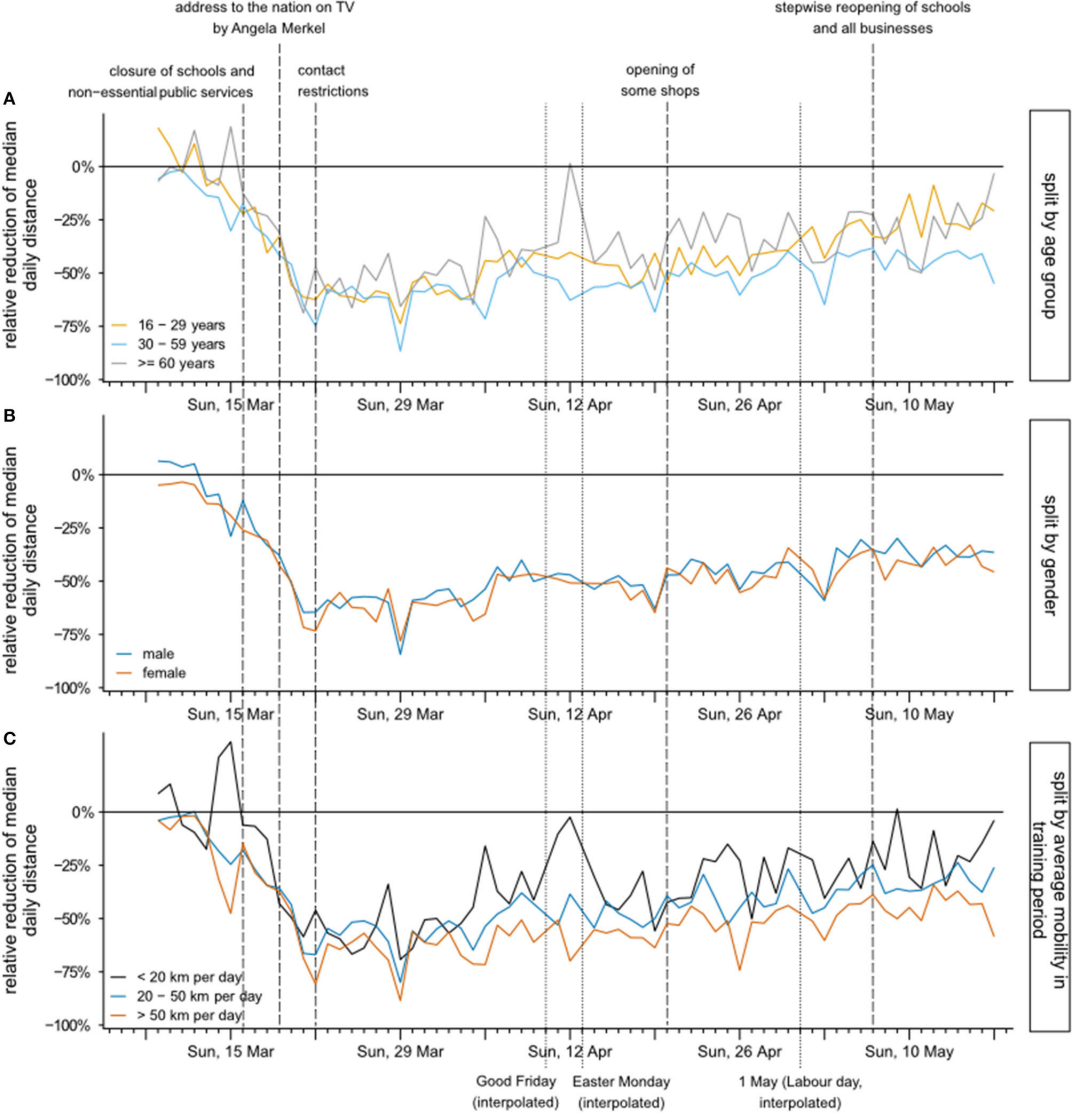
Relative reduction of median daily distances between 9 March and 17 May, stratified by age group **(A)**, gender **(B)**, and previous mobility defined by distances traveled between 13 January and 8 March **(C)**. Dates of important governmental interventions in Germany are indicated with dashed vertical lines.

Using the simulated data, we were able to calculate relative reductions of median daily distances traveled. In Figure 3, the relative reduction of median daily distance between 9 March and 17 May is displayed stratified by age group, gender, and previous travel habits. The relative reduction is remarkably similar in all age groups until Good Friday (10 April). After that day, individuals 60 years and older show a lower relative reduction, with a sharp increase in median daily distances on Easter Sunday (Figure 3A). However, this group has still by far the lowest absolute travel distance. No difference between genders can be observed regarding the relative reduction (Figure 3B). Interestingly, the relative reduction of median daily distance stayed comparable independent of previous travel habits until 4 April (Figure 3C). Afterwards, the relative reduction in the group with <20 km per day before mobility restrictions were implemented was smaller compared to the other groups, again with an outlier on Easter. In the days between 22 March and 4 April, the median relative reduction was as high as 50–75%. In the lockdown period thereafter (between 4 and 24 April), the reduction was smaller, mainly between 20 and 60%. In the period after easing the restrictions the mobility increased, however, remained below the values before the pandemic.

**Figure 4:**
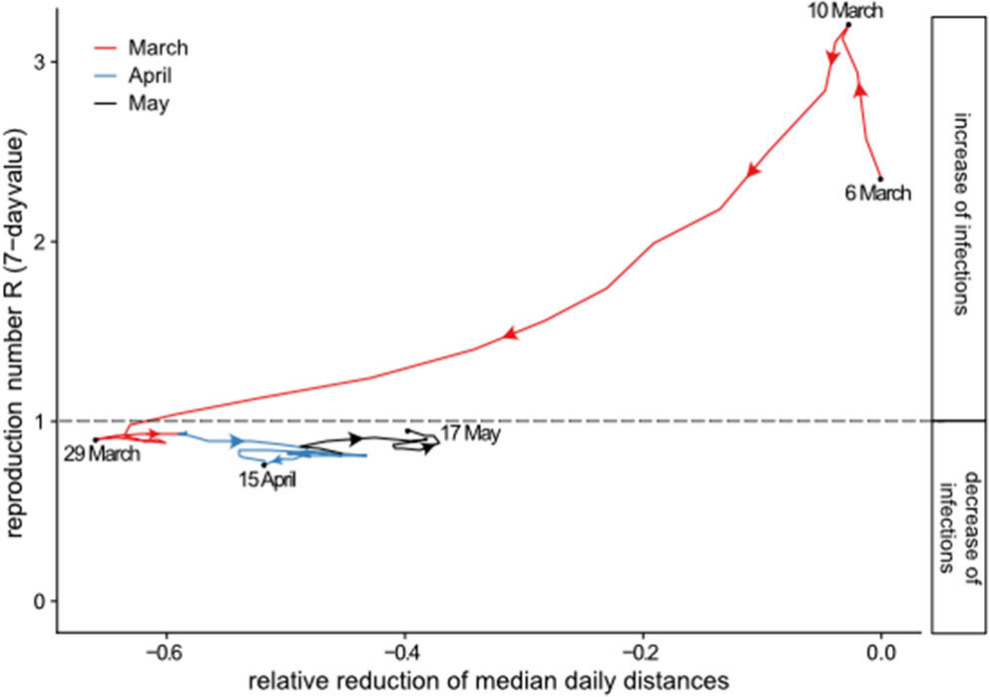
Relative mobility reduction vs. reproduction number R (7-day value) from 6 March to 17 May 2020.

To investigate the potential effect of the achieved mobility reduction on the spread of the virus, we consider the daily relative reduction against the daily reported number of cases and the estimate of the reproduction number for the period from 6 March to 17 May. Table 2 gives the daily number of cases (point estimate), the estimate of the reproduction number R (7-day value), and the relative mobility reduction smoothed by calculating a centered 7-day moving average^2^. A sharp reduction in mobility was observed in the period March 7 to March 20, followed by a short period of rather constant reduction of about 60% up to ~April 4, and thereafter a slow increase until the end of the observation period, with the reduction still being about 40%. The development of the daily case numbers, on the other hand, showed a different pattern. A strong increase in daily case numbers was seen from beginning of March for about 2 weeks. The peak period with ~5,000 cases per day on average was observed from March 16 to March 21, and after that an almost linear decline followed. The slope was stronger for 1 month until about April 21 with an average daily reduction of case numbers of 100. After that date, the decline gradually became smaller, resulting in daily numbers of about 500 cases per day toward the end of the observation period. The reproduction number was high (above 2) in the first half of March and decreased strongly to values below one thereafter, reaching a relatively constant value which varied between 0.76 and 0.92 after 23 March. This is further highlighted in Figure 4, which plots the relative reduction of mobility against R for each day of our study period. After a short period with increasing R and decrease of mobility from March 6 to 10, we observe a parallel decrease from 10 March until 28 March. After that date, as highlighted by color for the months April and May, the mobility increased but R remained on a relatively constant level. We conclude that the reduction on mobility, possibly in combination with other measures had a positive effect to reduce viral transmission. The increase in mobility thereafter was not followed by an increase of R which may be caused by other measures, such as general awareness, wearing masks, and others.

**Table 2:** Relative mobility reduction (7-day moving average), daily number of cases (smoothed point estimate), and reproduction number R (point estimate, 7-day value), 6 March to 17 May 2020, Germany.

**Date**	**Mobility^a^**	***N*^b^**	***R*^b^**
03/06/2020	−0.01	510	2.35
03/07/2020	−0.01	677	2.57
03/08/2020	−0.02	898	2.94
03/09/2020	−0.03	1,277	3.13
03/10/2020	−0.03	1,729	3.21
03/11/2020	−0.04	2,292	3.11
03/12/2020	−0.06	2,859	2.84
03/13/2020	−0.10	3,448	2.50
03/14/2020	−0.14	3,916	2.18
03/15/2020	−0.19	4,275	1.99
03/16/2020	−0.23	4,879	1.74
03/17/2020	−0.29	5,099	1.56
03/18/2020	−0.36	5,313	1.40
03/19/2020	−0.43	5,329	1.24
03/20/2020	−0.51	5,155	1.13
03/21/2020	−0.58	4,952	1.04
03/22/2020	−0.61	4,581	0.98
03/23/2020	−0.63	4,684	0.92
03/24/2020	−0.62	4,375	0.89
03/25/2020	−0.61	4,370	0.90
03/26/2020	−0.62	4,420	0.88
03/27/2020	−0.63	4,166	0.90
03/28/2020	−0.64	4,117	0.91
03/29/2020	−0.65	3,829	0.90
03/30/2020	−0.63	3,923	0.92
03/31/2020	−0.62	3,787	0.93
04/01/2020	−0.60	3,827	0.93
04/02/2020	−0.59	3,941	0.94
04/03/2020	−0.58	3,776	0.93
04/04/2020	−0.58	3,633	0.92
04/05/2020	−0.56	3,294	0.89
04/06/2020	−0.53	3,197	0.89
04/07/2020	−0.51	3,027	0.87
04/08/2020	−0.49	2,991	0.85
04/09/2020	−0.48	2,987	0.83
04/10/2020	−0.48	2,732	0.80
04/11/2020	−0.49	2,471	0.81
04/12/2020	−0.49	2,243	0.79
04/13/2020	−0.50	2,049	0.79
04/14/2020	−0.51	1,957	0.78
04/15/2020	−0.52	1,937	0.76
04/16/2020	−0.52	1,888	0.78
04/17/2020	−0.53	1,824	0.80
04/18/2020	−0.53	1,695	0.81
04/19/2020	−0.53	1,539	0.84
04/20/2020	−0.51	1,493	0.84
04/21/2020	−0.49	1,419	0.83
04/22/2020	−0.47	1,387	0.83
04/23/2020	−0.46	1,378	0.81
04/24/2020	−0.47	1,273	0.82
04/25/2020	−0.48	1,188	0.83
04/26/2020	−0.49	1,087	0.82
04/27/2020	−0.49	1,045	0.82
04/28/2020	−0.47	992	0.81
04/29/2020	−0.46	956	0.81
04/30/2020	−0.45	965	0.81
05/01/2020	−0.46	890	0.82
05/02/2020	−0.47	836	0.85
05/03/2020	−0.47	810	0.86
05/04/2020	−0.46	801	0.89
05/05/2020	−0.43	805	0.91
05/06/2020	−0.40	809	0.89
05/07/2020	−0.39	795	0.90
05/08/2020	−0.39	740	0.89
05/09/2020	−0.40	681	0.88
05/10/2020	−0.40	634	0.87
05/11/2020	−0.41	623	0.85
05/12/2020	−0.40	608	0.85
05/13/2020	−0.39	607	0.84
05/14/2020	−0.38	597	0.88
05/15/2020	−0.38	601	0.92
05/16/2020	−0.38	585	0.92
05/17/2020	−0.39	549	0.95

a *Relative mobility reduction, total cohort, 7-day moving average*.

b *Daily number of cases N (point estimate) and reproduction number R (7-day value) from: https://www.rki.de/DE/Content/InfAZ/N/Neuartiges_Coronavirus/Projekte_RKI/Nowcasting.html?nn=13490888*.

## Discussion

In this study, we showed a rapid decline in mobility in the middle of March after mobility restrictions were implemented, while by beginning of April, mobility increased again slightly. Reduction rates were considerable (Table 1). Mobility was more than halved in the lockdown period which demonstrates that lockdown politics of the government was respected by the population that obviously relinquished accustomed way of living. Even after relaxation of the strict lockdown measures from the end of April onwards, the mobility of the German population did not immediately go back to the pre-intervention level. The mobility reduction rate during the relaxation period (26 April−10 May) still reached about two-thirds of the reduction rate during lockdown (23 March−5 April). This demonstrates the continuation of the careful behavior of people even though political pressure was noticeably retracted. The relative reductions were found to differ little between age groups, gender, and groups with different mobility before the pandemic (Table 1, Figure 3). Also, the mobility reduction following the lockdown measures were found to be highly significant (*p* < 0.001) through all stratifications considered (age, gender, and region). On the other hand, no evidence for statistically significant period-by-subgroup interactions was found (large *p*-values), suggesting that the German population reduced its mobility in a rather consistent and uniform way.

We also examined the development of the reproduction number R which showed a parallel decline after March 10, a few days after the decline in mobility started, until the end of March. Afterwards, the mobility started to increase again, with R remaining stable below one.

This is the first study in Germany in the context of COVID-19 which combines mobility data with individual characteristics and which adds to a number of recent contributions on mobility changes following national mobility and contact restrictions [e.g., (16, 17)]. Analysis of Chinese mobility data suggests that restrictions were highly effective in reducing mobility and containing the spread of COVID-19 (18). Following the lockdown in Italy, Pepe et al. (19) reported a 50% reduction in mobility within and between provinces measured using large-scale anonymized location data from smartphones. The observed relative reduction in our study fall in the same range. Engle and colleagues combine aggregated mobility, infection, and demographic data at the US County level (20). They estimate that an official stay-at-home restriction reduces average mobility by 7.87%, which is far less reduction compared to our finding in Germany.

Apart from detailed descriptions of mobility behavior, we provide new evidence on the association between confirmed COVID-19 cases, reproduction number, and individual mobility. The decrease of mobility is rather a surrogate measure for transmission probability than a causal factor for the decrease in case numbers and reproduction number. The fact that the observed increase in mobility in April and May was not followed by an increase of the pandemic indicates that other behavioral changes may have played a major role. Yet, mobility can affect virus transmission dynamics by altering the frequency of contacts between infected and susceptible individuals from different households. Empirical research consistently finds a strong correlation between mobility and the spread and magnitude of various infectious diseases (21, 22). For COVID-19, Kraemer et al. (18) showed that mobility data recording travel in and out of Wuhan predicted very well the total number of cases outside of Wuhan during the early phase of the epidemic.

Our data have several advantages. First, the high spatial and temporal resolution of our tracking data enabled us to study individual mobility patterns at a higher precision than typically achieved with coarse-grained aggregated data from telecommunication or social media providers. Second, our panelists stem from a professionally managed and population-representative panel and explicitly agreed to be tracked for research purposes and voluntarily contributed their profile and location data. This enabled us to conduct detailed analyses of mobility patterns for selected stratifications, to study the influence of socio-demographic predictors on the outcome variable in detail, and to examine heterogeneity in behavior responses. This is in contrast with most other mobile data collection processes.

Following the Interrupted Time Series paradigm, we fitted a seasonal autoregressive time series model to the median daily distances during the reference period and produced mobility forecasts for the evaluation period. These forecasts play the role of a (non-existing) control group not exposed to mobility and contact restrictions, and thus represent the counterfactual scenario from which we deduced relative mobility reduction values. For the time-series analysis we chose the SARIMA (1, 0, 0) × (2, 0, 0, 7) model, as it showed a good fit and very satisfactory residual diagnostics while being parsimonious enough to avoid overfitting. The SARIMA model captures very well the weekly mobility patterns observed in the pre-intervention period and produces a stable and plausible counterfactual scenario. It could be argued that forecasting the mobility in April and May with a time series model that has been fitted to the data from January to mid-March possibly neglects weather-related effects. However, including such effects would considerably complicate the model without creating additional insights. If anything, including weather effects would further increase the relative mobility reductions obtained in our analysis.

Limitations of our analyses are the preliminarily restricted length of the time series and the possible selection bias due to the structure of the online panel. We are aware that our sample cannot be considered as a representative sample of the population. On the other hand, while our sample may not allow the unbiased estimation of the absolute mobility pattern in the German population, we consider it unlikely that the changes of mobility as observed in our sample are different. This is similar to the reasoning in a cohort study where the observed effect estimate of a factor on the disease risk may be unbiased even if the prevalence of the factor is smaller or larger than in the target population. Furthermore, reduced mobility is only a proxy for the reduction of social contacts and no causal prove for reduced viral transmission. However, the observed reduced mobility was parallel to the general rules to restrict meetings in public and in privacy with other people, to keep distance, and to take sanitary measures like frequent handwashing. Valid prediction models have to include further measures of behavior changes.

It remains to be seen how the course of the pandemic, the restrictions on mobility, and the behavior of the population will develop further. Ongoing analyses may help to select or develop effective measures adapted to different target populations.

## Data Availability Statement

The raw data supporting the conclusions of this article will be made available by the authors, without undue reservation.

## Ethics Statement

Ethical review and approval was not required for the study on human participants in accordance with the local legislation and institutional requirements. Written informed consent for participation was not required for this study in accordance with the national legislation and the institutional requirements.

## Author Contributions

SB analyzed the data and calculated the models. KW supervised and conceptualized statistical modeling and interpreted the results. LK interpreted the results, created the figures, and wrote the manuscript. SS and AZ interpreted the results. SW performed literature research and wrote the manuscript. HB and SM had the study idea and conceptualized the study. All authors contributed to writing the manuscript.

## Conflict of Interest

SB and SM are employed by the company “GIM Gesellschaft für Innovative Marktforschung mbH.” The remaining authors declare that the research was conducted in the absence of any commercial or financial relationships that could be construed as a potential conflict of interest.

## Footnotes

^1^Available online at: https://www.med.uni-muenchen.de/ethik/wann_beratungspflichtig.pdf (accessed July 14, 2020).

^2^Available online at: https://www.rki.de/DE/Content/InfAZ/N/Neuartiges_Coronavirus/Projekte_RKI/Nowcasting.html (accessed May 28, 2020).
